# Periprosthetic joint infection risk is comparable between unicompartmental knee arthroplasty to total knee arthroplasty conversion and primary total knee arthroplasty: A systematic review and meta‐analysis

**DOI:** 10.1002/ksa.70228

**Published:** 2025-12-08

**Authors:** Maximilian Budin, Matthew De Ruyter, Zachary Christopher Lum, Jürgen Slapar, Jochen Gerhard Hofstaetter, Georg Hauer, Andreas Leithner, Patrick Sadoghi

**Affiliations:** ^1^ Department of Orthopaedics and Trauma Medical University of Graz Graz Austria; ^2^ Department of Orthopaedic Surgery University of California Davis Medical Center Sacramento California USA; ^3^ Department of Orthopedic Surgery University of Missouri Kansas City Missouri USA; ^4^ Michael Ogon Laboratory for Orthopaedic Research Orthopaedic Hospital Vienna‐Speising Vienna Austria; ^5^ II Department of Orthopaedic Surgery Orthopaedic Hospital Vienna‐Speising Vienna Austria

**Keywords:** conversion UKA, knee arthroplasty revision, TKA, unicompartmental knee arthroplasty (UKA)

## Abstract

**Purpose:**

Unicompartmental knee arthroplasty (UKA) offers benefits over total knee arthroplasty (TKA), but some studies indicated higher revision rates, often involving conversion to TKA. The infection risk associated with UKA to TKA conversion compared to primary TKA is not definitively established. This systematic review and meta‐analysis aimed to determine if patients undergoing UKA to TKA conversion had a higher rate of periprosthetic joint infection (PJI) compared to primary TKA.

**Methods:**

Following preferred reporting items for systematic reviews and meta‐analysis guidelines, PubMed, EMBASE and SCOPUS were searched for clinical studies comparing PJI rates in adult patients undergoing UKA to TKA conversion versus primary TKA. Data on reoperations and component use were also extracted. A random‐effects model was used for the meta‐analysis.

**Results:**

Six retrospective studies involving 456 UKA to TKA conversion and 719 primary TKAs were included. No significant difference in the rate of PJI was found between the UKA to TKA conversion cohort (0.66%) and the primary TKA cohort (0.70%) (odds ratio [OR] 1.21, 95% confidence interval [CI] [0.27, 4.46]; *p* = 0.91). However, UKA to TKA conversion was associated with a significantly higher likelihood of requiring augments (15.6% vs. 3.9%; OR 8.71, 95% CI [1.29, 58.80]; *p* = 0.03) and stems (36.6% vs. 1.7%; OR 45.83, 95% CI [9.53, 220.55]; *p* < 0.00001), indicating greater surgical complexity.

**Conclusion:**

Based on current literature, UKA to TKA conversion is associated with a similar surgical site infection/PJI rate compared to primary TKA. UKA to TKA conversion procedures necessitated significantly more revision‐specific components.

**Level of Evidence:**

Level III.

**Abbreviations**
BMI: body mass index
OR: odds ratio
PICOS: Population, Intervention, Comparison, Outcome, Study design
PJI: periprosthetic joint infection
PRISMA: preferred reporting items for systematic reviews and meta‐analysis
ROBINS‐I: risk of bias in nonrandomised studies—of interventions
ROM: range of motion
TKA: total knee arthroplasty
UKA: unicompartmental knee arthroplasty

## BACKGROUND

Unicompartmental knee arthroplasty (UKA) is an established alternative to total knee arthroplasty (TKA) for single‐compartment osteoarthritis. UKA offers several potential benefits compared to TKA, which have been reported in various combinations across the literature, including faster postoperative recovery [19], enhanced proprioception [3], reduced postoperative pain [5], improved functional outcomes [13], greater range of motion [13] and lower reported rates of infection [6, 13]. Despite these advantages, registry data indicate a higher revision rate for UKA compared to TKA [15].

UKA revision typically involves the conversion to TKA. UKA failure is multifactorial, primarily involving aseptic loosening and progressive osteoarthritis in adjacent compartments [1, 22]. Persistent/intractable pain typically serves as the key clinical manifestation prompting revision [17]. While revision TKA procedures generally have a higher infection risk than primary TKA, the specific periprosthetic joint infection (PJI) risk following UKA to TKA conversion remains uncertain [7, 18]. It is unclear whether the PJI risk for UKA to TKA conversion aligns more with primary TKA or revision TKA. Understanding this specific risk is essential for preoperative patient counselling, surgical planning and postoperative management.

Individual retrospective studies exist, but no systematic review and meta‐analysis have aggregated this data to provide a more robust statistical estimate of the true infection risk of UKA to TKA conversion. This systematic review and meta‐analysis aimed to determine whether the rate of PJI is significantly higher in patients undergoing UKA to TKA conversion compared to those undergoing primary TKA. Based on the higher complexity of revision surgery, it was hypothesised that the UKA to TKA conversion group would have a significantly higher rate of PJI than the primary TKA group. A secondary objective was to assess the utilisation frequency of revision‐specific components such as cones and stems.

## METHODS

This review was designed, performed and reported in accordance with the preferred reporting items for systematic reviews and meta‐analyses (PRISMA) 2020 guidelines.

### Search strategy and study selection

The search strategy was structured according to the population, exposure, comparison, outcome, study design framework. The population (P) encompassed adult patients who had undergone a primary UKA (medial, lateral) and required conversion to a TKA. The exposure (E) was a conversion of a failed UKA to a TKA. For the comparison (C), studies with a comparator group of patients undergoing primary TKA were considered. The primary outcome (O) was defined as the incidence of PJI in the UKA to TKA conversion group compared to the primary TKA group. Secondary outcomes were defined as the use of additional fixation (e.g., stems, augments, sleeves and cones) during UKA to TKA conversion. Only clinical studies, cohort studies (both prospective and retrospective) and case‐control studies were considered (study design S). Case reports, reviews, editorials and letters to the editor were excluded. All English‐language articles identified through the search strategy that met the predefined inclusion criteria were included in the initial screening phase.

**Figure 1:**
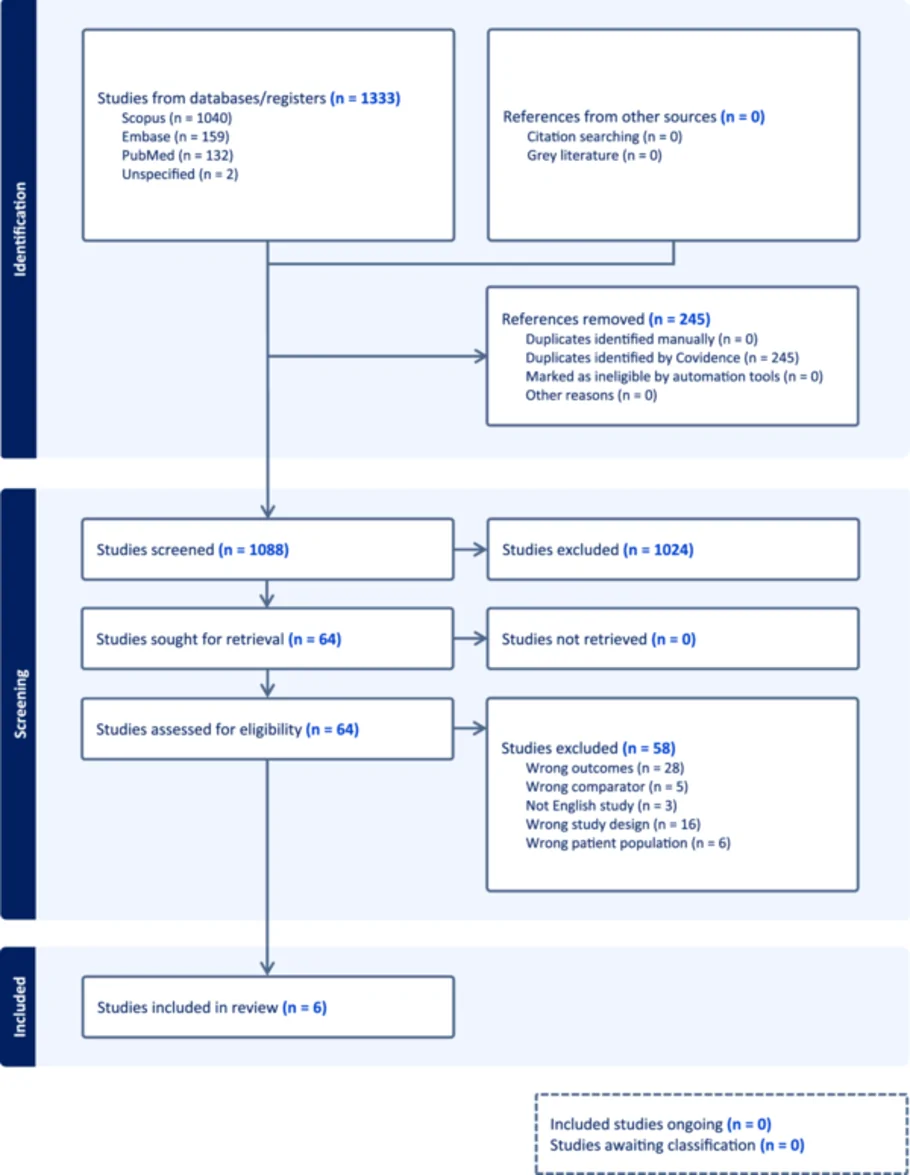
Preferred reporting items for systematic reviews and meta‐analysis (PRISMA) flow diagram illustrating the literature search and study selection process.

A comprehensive literature search was performed across PubMed, EMBASE and SCOPUS databases using the Covidence platform to identify relevant studies. In collaboration with a medical librarian, MeSH terms and the following keywords were utilised to search for literature: ‘unicompartmental knee arthroplasty’, ‘total knee arthroplasty’ and ‘periprosthetic joint infection’. This search methodology was adapted as necessary for each specific database. The reference lists of all included studies were manually reviewed to identify further relevant publications. All English‐language articles identified through the search strategy that met the predefined inclusion criteria were included in the initial screening phase. A full list of inclusion and exclusion can be found in Figure 1.

### Data extraction

Two independent reviewers, who were blinded to each other's decisions, screened titles and abstracts of all records to identify eligible studies. Any disagreements were resolved by consensus or by a third reviewer. A high level of initial agreement was found, with all disagreements resolved to 100% consensus. Following the selection of studies and confirmation of their relevance through full‐manuscript review, three reviewers independently extracted data from each included study to minimise extraction errors and to ensure accuracy. The primary data extracted concerned infection‐related complications in patients undergoing UKA to TKA conversion versus primary TKA. The secondary objectives were to determine the use of augments or stems during the revision surgery. Patient demographics, including age, gender and BMI, were recorded. The time interval from primary UKA to UKA to TKA conversion and the total follow‐up duration were noted. The level of evidence for each study was assigned based on established hierarchies. The risk of bias was assessed using the risk of bias in nonrandomised studies—of interventions tool.

### Statistical analysis

Data were summarised as dichotomous variables and odds ratios (ORs) with 95% confidence intervals (CIs). Dichotomous data were organised into 2 × 2 contingency tables to compute ORs, 95% CIs, effect sizes and standard errors consistently across studies. A meta‐analysis was performed using the systematic review and meta‐analysis tool RevMan 5.4.1. A random‐effects model was employed for the meta‐analysis to account for anticipated between‐study heterogeneity, potentially stemming from differences in study populations or methodologies. The analysis of secondary outcomes, including the utilisation rates of revision‐specific components (stems, augments and cones), was descriptive. Data were aggregated and are reported as absolute numbers (*n*) using Microsoft Excel 2021.

## RESULTS

Following a systematic literature search and screening process, a final cohort of six retrospective studies was included in this meta‐analysis. These studies specifically compared outcomes for patients undergoing UKA to TKA conversion against a control group undergoing primary TKA. The analysis encompassed a total of 456 patients in the UKA to TKA conversion cohort and 719 patients in the primary TKA cohort. All included studies were classified as providing level 3 or level 4 evidence [2, 4, 10, 11, 12, 14] (Table 1).

**Table 1:** Characteristics of included studies.

Characteristic	Cross 2014	Järvenpää 2010	Lim 2017	Lunebourg 2015	Lim 2019	Lombardi AV Jr 2018	Total
Study and cohort size							
Conversion UKA (*n*)	49	21	75	48	70	193	456
Primary TKA (*n*)	97	28	217	48	140	189	719
PJI outcomes							
PJI in conversion UKA (*n*)	1	0	0	1	0	1	3
PJI in primary TKA (*n*)	2	0	2	0	0	1	5
Follow‐up							
Follow‐up (months, mean)	57.6	126	62.4	84	73.2	73.2	
Follow‐up (months, range/SD)	24–204	96–204	38.4	48	24–165.6	24–240	
Patient demographics							
Mean age (conversion UKA)	61.5	74.9	65.6	71	65.2	63.5	
Mean age (primary TKA)	58.9	75.2	64.5	72	66.7	65.7	
Female % (conversion UKA)	61.2	57.1	81.3	75	82.9	59.1	
Female % (primary TKA)	‐	60.7	81.3	66.7	82.9	59.1	
Mean BMI (conversion UKA)	31.7	28.5	29.0	28.0	29.3	32.3	
Mean BMI (Primary TKA)	32.8	30.5	28.9	28.0	27.0	27.0	
Surgical characteristics							
Time to conversion (months, mean)	53.9	64.8	78	108	73.2	57.6	
Augment use (conversion UKA, *n*)	10	1	5	14	5	36	71
Augment use (primary TKA, *n*)	2	‐	25	0	1	‐	28
Stem use (conversion UKA, *n*)	15	6	37	35	33	41	167
Stem use (Primary TKA, *n*)	8	‐	1	0	3	‐	12

Abbreviations: BMI, body mass index; PJI, periprosthetic joint infection; TKA, total knee arthroplasty; UKA, unicompartmentla knee arthroplasty.

### PJI outcomes

**Figure 2:**
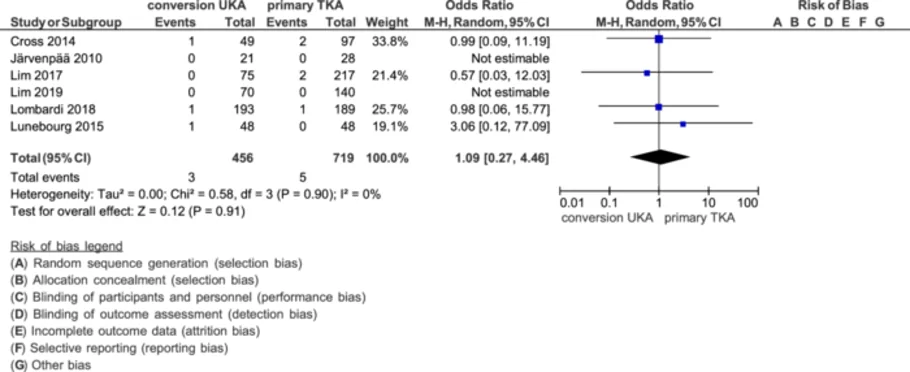
Forest plot comparing the odds of periprosthetic joint infection (PJI) in conversion unicompartmental knee arthroplasty (UKA) versus primary total knee arthroplasty (TKA). CI, confidence intervals.

The primary outcome was the incidence of PJI. In the UKA to TKA conversion cohort, 3 of 456 patients (0.66%) experienced a PJI, compared to 5 of 719 patients (0.70%) in the primary TKA cohort. A meta‐analysis confirmed no statistically significant difference in the odds of PJI between the groups (OR 1.09, 95% CI [0.27, 4.46], *p* = 0.91). The analysis showed no heterogeneity between studies (*I*² = 0%) (Figure 2).

### Patient demographics

The demographic characteristics were broadly similar between the two groups across the included studies, suggesting comparable baseline profiles. The mean age for UKA to TKA conversion group ranged from 61.5 to 74.9 years, compared to a range of 58.9–75.2 years for the primary TKA group. The proportion of female patients was also consistent, ranging from 57.1% to 82.9% in the UKA to TKA conversion cohort and 60.7%–82.9% in the primary cohort. The mean body mass index (BMI) did not differ substantially, with ranges of 28.0–32.3 kg/m² for UKA to TKA conversion patients and 27.0–32.8 kg/m² for primary TKA patients (Table 1).

### Surgical characteristics and follow‐up

**Figure 3:**
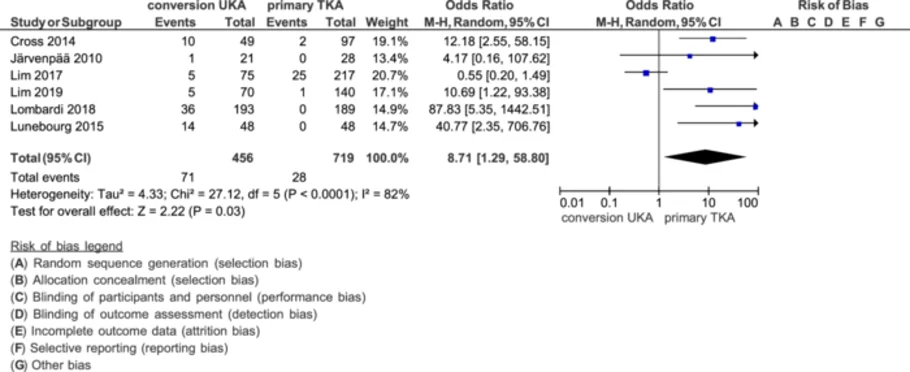
Forest plot comparing the odds of requiring augments in conversion unicompartmental knee arthroplasty (UKA) versus primary total knee arthroplasty (TKA). CI, confidence intervals.

While PJI rates were similar, a notable difference was observed in surgical complexity. The requirement for both augments and stems was substantially higher in the UKA to TKA conversion cohort. Specifically, 71 of 456 (15.6%) UKA to TKA conversion patients required augments, compared to only 28 of 719 (3.9%) in the primary TKA group. This difference was statistically significant, with the odds of requiring augments being nearly nine times higher in the UKA to TKA conversion group (OR 8.71, 95% CI [1.29, 58.80], *p* = 0.03) (Figure 3).

**Figure 4:**
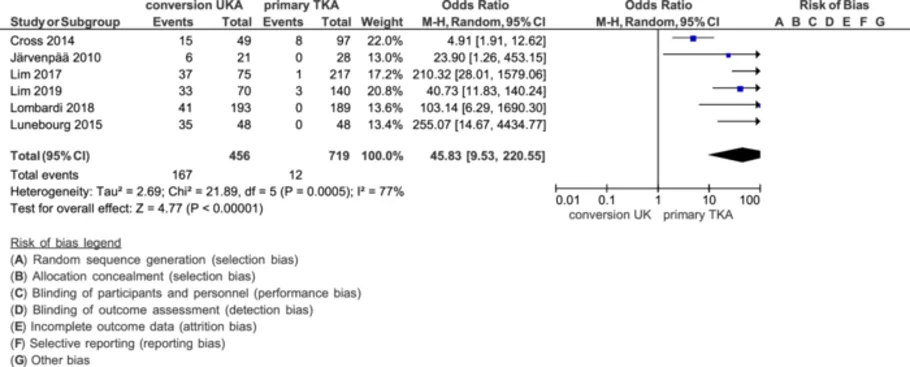
Forest plot comparing the odds of requiring stems in conversion unicompartmental knee arthroplasty (UKA) versus primary total knee arthroplasty (TKA). CI, confidence intervals.

Similarly, 167 of 456 (36.6%) UKA to TKA conversion patients required stems, versus just 12 of 719 (1.7%) primary TKA patients. The meta‐analysis revealed that the odds of requiring stems were over 45 times greater in the UKA to TKA conversion cohort (OR 45.83, 95% CI [9.53, 220.55], *p* < 0.00001). Significant heterogeneity was observed in the analyses for both augments (*I*² = 82%) and stems (*I*² = 77%). The mean time from the index UKA to the UKA to TKA conversion procedure ranged from 53.9 to 108 months, and the mean follow‐up period across studies varied from 57.6 to 126 months (Table 1, Figure 4).

## DISCUSSION

The most important finding of the present study was that UKA to TKA conversion was associated with a similar rate of PJI compared to primary TKA. PJI rates observed in the UKA to TKA conversion cohort were consistent with previously reported rates for primary TKA in the literature [16, 23]. This finding is notable because the conversion procedures were demonstrably more complex, with a significantly higher use of stems and cones.

Several factors might explain the lack of an elevated infection risk in the UKA to TKA conversion. The relatively low absolute number of surgical site infection (SSI)/PJI events reported across the included studies could limit the statistical power to detect a true difference. A majority of the studies included in this review were from practice registries. Such registries often originate from high‐volume centres with surgeons who possess considerable expertise in both UKA and complex revision arthroplasty. This expertise bias or center effect could lead to lower complication rates, including infections, than those that might be observed in a more generalised population of surgeons and centres [9]. Database studies, which capture a broader range of surgical experiences, including lower‐volume centers, might yield different findings.

Despite comparable infection rates between both groups in this study, a notable finding of this review was the significantly higher use of augments, such as stems and augments, during UKA to TKA conversion. This is often necessitated by bone loss encountered at the site of the failed UKA, particularly on the tibial side, or to achieve adequate stability and fixation. Previous studies highlighted that an increased polyethylene bearing thickness in the primary UKA, often a surrogate for greater tibial bone resection, correlated with a higher likelihood of requiring stems and augments during UKA to TKA conversion [12, 20, 21, 24]. This underscores the importance of preoperative planning and intraoperative assessment for bone defects and the potential need for these adjunctive components. Lewis et al. suggested that the prophylactic use of tibial stems during UKA to TKA conversion might be associated with a lower subsequent revision risk, suggesting the use of these modes of fixation may be beneficial [8]. While this increased use of revision‐specific components did not correlate with a higher PJI rate in this analysis, it is plausible that the increased surgical time, complexity and implant surface area associated with these components could introduce a confounding risk of infection that warrants further study.

This systematic review and meta‐analysis had several limitations that should be acknowledged. The restriction to English‐language articles might have excluded relevant studies from non‐English‐speaking regions, potentially introducing publication bias. The predominance of practice registry studies, as previously discussed, may limit the generalisability of our findings due to potential expertise bias. Large database studies are needed to confirm these results in a broader patient and surgeon population. Underreporting of SSIs/PJIs or other complications in registry data is always a concern. The low absolute number of infections also affected the precision of our estimates. Capturing true ‘UKA to TKA conversion’ procedures in large databases can be challenging due to coding limitations, potentially leading to misclassification and/or underestimation. Based on the limited and underpowered current literature, no statistically significant difference in the rate of PJI was detected between UKA‐to‐TKA conversion and primary TKA. This finding must be interpreted with extreme caution, as the low number of PJI events provides insufficient statistical power to rule out a clinically important difference. Due to the limited number of studies and patients within specific subgroups, robust subgroup analyses could not be performed to determine whether PJI rates differed significantly between types of UKAs (e.g. fixed‐bearing vs. mobile‐bearing, medial vs. lateral). A potential limitation is the inclusion of various failure etiologies. However, conversion for periprosthetic fracture was exceedingly rare, with only six cases identified across all studies. While these fractures typically necessitate complex reconstruction with augments and stems, this low number indicates that the observed need for such implants in the UKA to TKA conversion is overwhelmingly attributable to bone loss and implant instability associated with the more common aseptic and mechanical failure modes.

## CONCLUSION

This meta‐analysis indicated as similar PJI rate in UKA to TKA conversion compared to primary TKA. This finding provides reassuring evidence for clinical practice and supports UKA as a viable treatment strategy. The findings on augment usage serve as a critical preoperative reminder. The significantly higher use of augments underscores the complexity of these revisions. Surgeons must preoperatively anticipate significant bone loss and be prepared for these technically demanding operations by ensuring a full inventory of revision components is available. It is important to note that the high utilisation of augments is not inherently negative. Their application is crucial for managing bone loss and ensuring stability. In fact, some evidence suggested that their application in UKA to TKA conversion can be associated with improved implant survivorship. Further research, particularly utilising large, comprehensive national databases, is warranted to confirm these findings, better identify risk factors for failure and explore strategies to optimise outcomes for all patients undergoing this common revision procedure.

## AUTHOR CONTRIBUTIONS

Maximilian Budin was responsible for the study design, data collection, formal analysis and the drafting of the initial manuscript. Jürgen Slapar contributed to the data analysis and participated in the critical revision of the manuscript. Patrick Sadoghi conceived the study, provided supervision and performed the final manuscript revision. All authors have read and approved the final manuscript.

## CONFLICT OF INTEREST STATEMENT

The authors declare no conflicts of interest.

## ETHICS STATEMENT

The authors have nothing to report.

## Supporting information

supporting information.

supporting information.

supporting information.

supporting information.

supporting information.

supporting information.

supporting information.

supporting information.

supporting information.

## Data Availability

The data supporting the findings of this study can be found within the original papers.
