# Supplementary material for: Periprosthetic joint infection risk is comparable between unicompartmental knee arthroplasty to total knee arthroplasty conversion and primary total knee arthroplasty: A systematic review and meta‐analysis
Source: Knee Surg Sports Traumatol Arthrosc. 2025 Dec 8;34(9):3314–21. doi: 10.1002/ksa.70228 (PMC13502457; doi:10.1002/ksa.70228)
Supplement: Supplementary file 1 [file KSA-34-3314-s001.docx]

The Risk Of Bias In Non-randomized Studies – of Interventions, Version 2 (ROBINS-I V2) assessment tool

(for follow-up studies)

**November 2024**

This work is licensed under a [Creative Commons Attribution-NonCommercial-NoDerivatives 4.0 International License](http://creativecommons.org/licenses/by-nc-nd/4.0/).

VERSION 2: LAUNCH VERSION, 22 November 2024

## Outline of ROBINS-I V2

ROBINS-I aims to assess the risk of bias in a specific result from an individual non-randomized study that examines the effect of an intervention on an outcome. This document describes the ROBINS-I V2 tool for **follow-up (cohort) studies**. Assessments should relate to risk of **material bias** rather than risk of any bias. Material bias should be interpreted as bias sufficient to cause an important change to the magnitude of the estimated effect, compared with the true value.

Before undertaking a ROBINS-I assessment (or series of assessments, e.g. in the context of a systematic review), users of the tool should specify the important confounding factors that are likely to influence the association between the intervention and the outcome (see section “At planning stage”).

The start point for an assessment of a specific study is to specify the result from the study that is being assessed for risk of bias. A ‘screening’ section then facilitates identification of results that are at “Critical risk of bias”, allowing the user to avoid a detailed assessment.

A key feature of the ROBINS-I approach is the specification, for each study, of the causal effect estimated by the result under consideration through specification of a hypothetical ‘target trial’. This is essential for assessment of risk of bias, because the causal effect defines the result that would be seen (other than the impact of sampling variation) in the absence of bias.

If multiple assessors will implement ROBINS-I independently, the *Preliminary considerations to plan the assessment* should be agreed between all assessors before each assessor works individually through evaluation of the confounding factors and bias domains.

ROBINS I includes seven domains of bias:

- Domain 1: Risk of bias due to confounding
- Domain 2: Risk of bias in classification of interventions
- Domain 3: Risk of bias in selection of participants into the study (or into the analysis)
- Domain 4: Risk of bias due to deviations from intended interventions
- Domain 5: Risk of bias due to missing data
- Domain 6: Risk of bias arising from measurement of the outcome
- Domain 7: Risk of bias in selection of the reported result

Each bias domain in ROBINS-I is addressed using a series of **signalling questions** that aim to gather important information about the study and the analysis being assessed. Most signalling questions have response options ‘Yes’, ‘Probably yes’, ‘Probably no’, ‘No’ and ‘No information’, with ‘Yes’ and ‘Probably yes’ having the same implications for risk of bias and similarly for ‘No’ and ‘Probably no’. Some questions have additional response options (a ‘weak’ and a ‘strong’ version of ‘Yes’ or ‘No’) to help discriminate between higher and lower risk of bias. After the relevant signalling questions have been completed, an algorithm maps the answers to the signalling questions onto a proposed judgement about **risk of bias** in the result that arises from this domain. The judgements and their broad interpretations are as follows.

| Judgement | Interpretation |
| --- | --- |
| *Low risk of bias** | There is little or no concern about bias with regard to this domain. |
| *Moderate risk of bias* | There is some concern about bias with regard to this domain, although it is not clear that there is an important risk of bias. |
| *Serious risk of bias* | The study has some important problems in this domain: characteristics of the study give rise to a serious risk of bias. |
| *Critical risk of bias* | The study is very problematic in this domain: characteristics of the study give rise to a critical risk of bias, such that and the result should generally be excluded from evidence syntheses. |

*For Domain 1 (Risk of bias due to confounding), this is referred to as “Low risk of bias (except for concerns about uncontrolled confounding)”, in which confounding is very well addressed but cannot be eliminated as a possibility. This is because a risk of bias due to uncontrolled confounding cannot be excluded in an observational study.

ROBINS-I is intended to provide a framework for making informed and reasonable judgements about risk of material bias in studies of the effects of intervention on outcome. On occasion, answers to the signalling questions may not yield an appropriate risk of bias judgement based on the algorithm. Therefore, suggested risk of bias judgements produced by the algorithms can be overridden, in which case justification should be provided. We aim for transparency and reasonableness rather than mechanistic adherence to every word of the tool’s contents.

Optionally, a **predicted direction of bias** may be selected, balancing the various issues addressed within the domain. Response options for this depend on the type of bias being addressed.

After completing all seven bias domains, an **overall judgement** is made for the risk of bias (and optionally for the predicted direction of any bias). The risk-of-bias Judgement is derived from the domain-level judgements using an algorithm. As for bias domain-level judgements, justification should be provided when the overall judgement suggested by the algorithm is overridden.

An online implementation of ROBINS-I V2 including automatic selection of relevant signalling questions and algorithm-derived risk-of-bias judgements is available via www.riskofbias.info.

# The ROBINS-I V2 tool

## At planning stage: list confounding factors

P1. List the important confounding factors relevant to all or most studies on this topic. Specify whether these are particular to specific intervention-outcome combinations.

#### Guidance notes

A confounding factor is a prognostic factor that predicts the interventions received. Important confounding factors are those that have the potential to introduce material bias into an estimated effect. Factors that are expected to have only very weak associations with the intervention or with the outcome, such that failure to account for them in the analysis will not have a material impact on the estimated effect of intervention on outcome, need not be considered here. Important confounding factors should be pre-specified at the planning stage, for example in the protocol of a systematic review that will include studies of the effects of interventions. The identification of potential confounding factors requires content knowledge and may usefully be informed by examination of relevant literature. Important confounding factors should be specified at the level of the broad research question (e.g. using a single list of confounding factors for a systematic review). This broad question may cover several specific interventions and/or outcomes. If confounding factors are specific to particular intervention-outcome combinations, then this should be stated.

### **Confounding Factors Relevant to This Study**

- **Age**: HTO is more commonly performed in younger patients, which may affect outcomes and patient expectations.
- **Sex**: Differences in pain perception and functional reporting between men and women can influence PROMs.
- **Body Mass Index (BMI):** UKA is less frequently chosen for patients with high BMI, which is itself associated with worse functional outcomes.
- **Comorbidities:** The presence of additional health conditions can influence both perceived quality of life and functional recovery.
- **Preoperative symptom severity / surgical indication**: HTO and UKA are often indicated for different stages or patterns of knee osteoarthritis, which affects baseline function and outcomes.
- **Time since surgery**: Differences in follow-up timing can influence outcome reporting and recovery assessment.
- **Center/surgeon volume**: Surgical expertise and hospital volume may influence both the selection of procedure and patient outcomes.

# For each study result: preliminary considerations

#### Guidance notes

The following questions should be answered only for the specific result that is being evaluated for the current ROBINS-I assessment.

In case of multiple alternative analyses being presented, it is important to specify the numeric result (e.g. RR = 1.52 (95% CI 0.83 to 2.77) and/or a reference (e.g. to a table, figure or paragraph) that uniquely defines the result being assessed.

Some characteristics of a study or a result may lead directly to the result being at critical risk of bias, and so make detailed risk-of-bias assessments unnecessary. A series of preliminary questions in this section aim to identify such situations.

Two preliminary questions are used to examine whether there is a need to examine time-varying confounding in the first domain of the tool (Bias due to confounding). If participants could switch between intervention groups then associations between intervention and outcome may be biased by time-varying confounding. This occurs when prognostic factors influence switches between intended interventions. For example, in a cohort study of the effect of antiretroviral therapy (ART) on rates of AIDS and death in people with HIV, follow-up time for each participant was split according to receipt of ART. Because CD4 counts during follow-up influenced the decision to start ART, CD4 count was a time-varying confounder.

The **target randomized trial specific to the study** is a hypothetical randomized trial, which need not be ethical or feasible, that compares the health effects of the same interventions, conducted with the same eligibility criteria as the non-randomized study. In general, such target trials will not use blinding of participants or of health professionals administering interventions.

**If multiple assessors will implement ROBINS-I independently, the questions in this section should be agreed between all assessors before each assessor works individually through the risk-of-bias assessment itself.**

## A. Specify the result being assessed for risk of bias

#### 2BGuidance notes (Specifying the numerical result)

A ROBINS-I assessment of risk of bias is specific to a particular study result. This is because different results from the same study may be at importantly different risks of bias (consider, for example, an unadjusted estimate of intervention effect compared with an estimate that is adjusted for numerous important confounding factors). Consequently, it may be necessary to undertake several ROBINS-I assessments of different results from the same study. If the study presents multiple alternative analyses, specify the numerical result (e.g. RR=1.52 (95% CI 0.83 to 2.77)) and/or a reference (e.g. to a table, figure or paragraph) that uniquely defines the result being assessed.

A1. Specify the numerical result being assessed

The primary numerical results are **patient-reported outcome scores** measured at 1 year postoperatively:

- **KOOS subscales** (Pain, Symptoms, ADL, Sport, QoL)
- **EQ-5D Index** (general health utility score)
- **VAS for pain**
- **Satisfaction score**

These results are reported as **mean scores ± standard deviation** and compared between HTO and UKA groups.

A2. Provide further details about this result (for example, location in the study report, reason it was chosen) [optional]

## B. Decide whether to proceed with a risk-of-bias assessment

#### 3BGuidance notes (Whether to proceed with a risk-of-bias assessment)

Some characteristics of a study or a result may lead directly to the result being at critical risk of bias, and so make detailed risk-of-bias assessments unnecessary. The questions in this section aim to identify such situations.

| **B1 Did the authors make any attempt to control for confounding?** | Confounding is a substantial problem in most non-randomized studies, and it is usually important to control for the important confounding factors. | Y |
| --- | --- | --- |
| **B2 If N/PN to B1: Is there sufficient potential for confounding that an unadjusted result should not be considered further?** | If there is sufficient potential for confounding that an unadjusted result should not be considered further, then the result is judged to be at ‘Critical risk of bias’. | N |
| **B3 Was the method of measuring the outcome inappropriate?** | This question aims to identify methods of outcome measurement (data collection) that are unsuitable for the outcome they are intended to evaluate. This enables a rapid assessment that a result should be regarded as at ‘Critical risk of bias’.  The question does not aim to assess whether the choice of outcome being evaluated was *sensible* (e.g. because it is a surrogate or proxy for the main outcome of interest). In most circumstances, for pre-specified outcomes, the answer to this question will be ‘N’ or ‘PN’.  Answer ‘Y or ‘PY’ if the method of measuring the outcome is inappropriate, for example because:   (1) important ranges of outcome values fall outside levels that are detectable using the measurement method; or (2) the measurement instrument has been demonstrated to have such poor reliability or validity that estimates of the relationship between intervention and the measured outcome are not useful. (3) The measurement method differed substantially between people in the intervention and comparator groups, so that differences between the groups are not interpretable. | N |

**If the answer to either B2 or B3 is ‘Yes’ or ‘Probably yes’, the result should be considered to be at ‘Critical risk of bias’ and no further assessment is required.**

## C. Specify the analysis in the current study for which results are being assessed for risk of bias

Specify the outcome to which this result relates

The result relates to patient-reported functional outcomes and health-related quality of life following either high tibial osteotomy (HTO) or unicompartmental knee arthroplasty (UKA). Specifically, the outcome includes the following validated instruments:

- **KOOS** (Knee injury and Osteoarthritis Outcome Score) subscales: Pain, Symptoms, Activities of Daily Living (ADL), Sport and Recreation, and Quality of Life (QoL)
- **EQ-5D Index Score**, measuring general health utility
- **VAS** (Visual Analog Scale) for pain
- **Satisfaction Score**, reflecting patient satisfaction with the surgical outcome

These outcomes are used to evaluate differences in subjective function, pain relief, and quality of life between the two intervention groups one year after surgery.

C1. Specify the participant group on which this result was based.

The result was based on a matched cohort of patients identified from the Swedish National Knee Arthroplasty Register (SKAR). Participants were adults who underwent either high tibial osteotomy (HTO) or unicompartmental knee arthroplasty (UKA) for medial knee osteoarthritis.

All participants completed validated patient-reported outcome measures (PROMs) one year after surgery, including the KOOS, EQ-5D, VAS for pain, and a satisfaction score.

*C2 to C3. Determine whether there is a need to consider time-varying confounding*

C2. Was the analysis based on splitting participants’ follow up time according to intervention received, or was follow-up censored when participants in one group switched to another group (e.g. when comparison group participants started the intervention)?

| x | No | Use Variant A of Domain 1 |
| --- | --- | --- |
| □ | Yes | Proceed to next question |

C3. If **Y** to C2, were intervention discontinuations or switches likely to be related to factors that are predictive of the outcome?

| □ | No | Use Variant A of Domain 1 |
| --- | --- | --- |
| □ | Yes | Use Variant B of Domain 1 |

## D. Specify a (hypothetical) target randomized trial specific to the study

#### Guidance notes

Evaluations of risk of bias are facilitated by considering the non-randomized study as an attempt to emulate a pragmatic randomized trial, which we refer to as the **target trial**. The first part of a ROBINS-I assessment for a particular study is to specify a target trial - the hypothetical randomized trial whose results should be the same as those from the non-randomized study under consideration, in the absence of bias. Its key characteristics are the types of participant (including exclusion/inclusion criteria) and descriptions of the intervention strategy and comparator strategy. These issues were considered in more detail by Hernán (2016). Differences between the target trial for the individual non-randomized study and the generic research question of the review relate to issues of heterogeneity and/or generalizability rather than risk of bias.

Because it is hypothetical, ethics and feasibility need not be considered when specifying the target trial. For example there would be no objection to a target trial that compared individuals who did and did not start smoking, even though such a trial would be neither ethical nor feasible in practice.

Selection of a patient group that is eligible for a target trial may require detailed consideration, and lead to exclusion of many patients. For example, Magid et al (2010) studied the comparative effectiveness of ACE inhibitors compared to beta-blockers as second-line treatments for hypertension. From an initial cohort of 1.6m patients, they restricted the analysis population to (1) persons with incident hypertension, (2) who were initially treated with a thiazide agent, and (3) who had one of the two drugs of interest added as a second agent for uncontrolled hypertension, and (4) who did not have a contraindication to either drug. Their “comparative effectiveness” cohort included 15,540 individuals: less than 1% of the original cohort.

*A note on terminology*: Throughout ROBINS-I V2, we refer regularly to “intervention” and “comparator”. The comparator may be an alternative active intervention, a control condition or no intervention at all.

We sometimes refer to the “intervention strategy” and “comparator strategy”, because an intervention typically consists of a package of care or procedures, and may be implemented over a period of time rather than on a single occasion. Specification of the whole strategy of interest is particularly important when interest is in a ‘per protocol’ effect.

In non-randomized studies, assignment to the intervention or comparator is inferred from the recorded intervention for each participant. This is in contrast to randomized trials, in which participants are randomly assigned to the intervention or comparator. We refer to the participants assigned to each strategy as the “intervention group” and “comparator group”.

Hernán MA, Robins JM. Using big data to emulate a target trial when a randomized trial is not available. *American Journal of Epidemiology* 2016;183:758-64; doi:10.1093/aje/kwv254.

Magid DJ, Shetterly SM, Margolis KL, Tavel HM, O’Connor PJ, Selby JV, Ho PM. Comparative effectiveness of angiotensin-converting enzyme inhibitors versus beta-blocker as second-line therapy for hypertension. *Circulation: Cardiovascular Quality and Outcomes* 2010;3:453-458; doi:10.1161/CIRCOUTCOMES.110.940874.

| D1. Specify the participants and eligibility criteria |  |
| --- | --- |
| D2. Specify the intervention strategy |  |
| D3. Specify the comparator strategy |  |

## E. Decide on the effect of interest

E1. Is your aim for this study…?

| □ | to assess the intention-to-treat effect (the effect of *assignment to* an intervention strategy or comparator strategy) |
| --- | --- |
| □ | to assess a per-protocol effect (the effect of *adhering to* a specified intervention strategy or comparator strategy) |

E2. **If the aim is to assess a per-protocol effect**, briefly define the changes to the intervention or comparator strategies that will be considered to be protocol deviations and, optionally, those changes that will not be considered. For example, the protocol deviations considered could be: “Starting intervention among comparator group participants, while acceptable changes could be “stopping intervention because of intervention-related toxicities occur or disease progression” or “changes to intervention after the trial baseline”.

# F. Information sources

#### Guidance notes

Evaluation of a study should be based on the maximum possible amount of available information. In addition to published papers describing a study’s methods and results, such information may be derived from the study protocol, unpublished reports or through correspondence with the study investigators.

Which of the following sources have you obtained to help you inform your risk of bias judgements (tick as many as apply)?

- Journal article(s)
- Study protocol
- Statistical analysis plan (SAP)
- Non-commercial registry record (e.g. ClinicalTrials.gov record)
- Company-owned registry record (e.g. GSK Clinical Study Register record)
- “Grey literature” (e.g. unpublished thesis)
- Conference abstract(s)
- Regulatory document (e.g. Clinical Study Report, Drug Approval Package)
- Individual participant data
- Research ethics application
- Grant database summary (e.g. NIH RePORTER, Research Councils UK Gateway to Research)
- Personal communication with investigator
- Personal communication with sponsor

Please specify any additional sources not listed above

# Evaluation of confounding factors

Complete a row for each important confounding factor listed in advance (subsection (i) below); and either relevant to the setting of this particular study or identified by the study authors (subsection (ii)). **“Important” confounding factors are those for which, in the context of this study,** **adjustment is expected to lead to a meaningful change in the estimated effect of the intervention.**

#### Guidance notes

Confounding is of fundamental importance to the analysis and interpretation of non-randomized studies of the effect of interventions on outcomes. ROBINS-I addresses two types of confounding: baseline confounding and time-varying confounding. **Baseline confounding** occurs when one or more prognostic factors, present before the start of the intervention, predict intervention received. Appropriate methods to control for confounders measured at baseline include stratification, regression, matching, standardization, and inverse probability weighting. The analysis may control for individual variables or for estimated propensity scores (inverse probability weighting is based on a function of the propensity score).

**Time-varying confounding** needs to be considered in studies that partition follow-up time for individual participants according to intervention received.

We use the term **confounding factor** for each broad source of potential confounding. It may not be possible to measure a factor well, and we distinguish between the confounding factor and the **variables** used to measure it. These variables may be used, for example, as covariates in a regression analysis.

In the context of a particular study, variables need not be included in the analysis: (a) if they are not associated with the outcome, conditional on intervention received (noting that lack of a statistically significant association is not evidence of a lack of association); (b) if they are not associated with intervention; (c) if adjustment makes no or minimal difference to the estimated effect of intervention on outcome; (d) because the confounder was addressed in the study design, for example by restricting to individuals with the same value of the confounder; (e) because a negative control demonstrates that there was unlikely to have been confounding due to this variable or that uncontrolled confounding was likely to be minimal; or (f) because external evidence suggests that controlling for the variable is not necessary in the context of the study being assessed.

In some studies, researchers may include a very large set of potential confounding variables in an analysis without considering their associations with outcome and intervention. Users of ROBINS-I should focus on (i) the confounding factors they determined a priori to be important and (ii) other factors for which adjustment is expected to lead to an important change in the estimated effect of the intervention on the outcome in the context of the current study.

Users of ROBINS-I should evaluate the confounding factors that they prespecified as important for the intervention-outcome relationship under study. The tool also allows the user to evaluate a second list of any further confounding factors that are either relevant to the setting of this particular study or which the study authors identified as potentially important. It is likely that new ideas relating to confounding and other potential sources of bias will be identified after the drafting of the review protocol, and even after piloting data collection from studies selected for inclusion in the systematic review. For example, such issues may be identified because they are mentioned in the introduction and/or discussion of one or more papers. This could be addressed in practice by explicitly recording whether potential confounders or other sources of bias are mentioned in the paper.

In very rare situations it is possible that no confounding factors are present, either because interventions received are known to be unrelated to any prognostic factors for the outcome of interest, or because no such prognostic factors exist. In such situations, the risk of bias due to confounding may be assessed as low.

The purpose of this preliminary assessment of confounding factors is to review the extent to which the result being assessed was controlled for confounding, considering both the prespecified confounding factors and any further confounding factors identified as important in the context of the study being assessed. This enables users of ROBINS-I to answer the signalling questions for the Domain 1 assessment (Risk of bias due to confounding). “Important” confounding factors are those for which, in the context of this study, adjustment is expected to lead to an important change in the estimated effect of the intervention.

The preliminary assessment consists of the following steps for each confounding factor.

- determine which variables (if any) were measured for the factor;
- determine which of these variables were controlled for in the analysis;
- for variables that were not controlled for, look for evidence that controlling for the variable was not necessary in this particular study;
- determine whether the confounding factor was measured validly and reliably by the variables used to measure it (this is assessed at the level of the confounding factor rather than the level of the individual variables used to measure the factor);
- determine the likely direction of bias if the analysis fails to adjust for this variable (alone).

The direction of bias, if the analysis fails to adjust for a particular variable (alone), will be that the effect estimate is biased *upwards* or biased *downwards*. For example, if older age predicts that a particular intervention is more likely to be received and the outcome is mortality, then this confounding would bias the estimated effect downwards: unless we adjust for age the intervention will appear more positively associated with higher mortality than it should. In the presence of *positive confounding* (the confounder is positively associated with both intervention and outcome, or negatively associated with both intervention and outcome), the bias will be upwards. In the presence of *negative confounding* (the confounder is positively associated with intervention and negatively associated with outcome, or vice versa), the bias will be downwards.

| **(i) Important confounding factors listed in advance** | | | | | | |
| --- | --- | --- | --- | --- | --- | --- |
| Confounding factor | Measured variable(s) for this factor, if any | Was this variable (or were these variables) controlled for in the analysis?  (Y / N) | If this confounding factor was controlled for, was it measured validly and reliably by this variable (or these variables)?*  (NA / Y / PY / PN / N / NI) | If this confounding factor was not controlled for, is there evidence that controlling for it was unnecessary?**  (NA / Y / PY / PN / N) | OPTIONAL: Is failure to adjust for this confounding factor expected to bias the effect estimate upwards or downwards? (Upward bias (overestimate the intervention effect) / Downward bias (underestimate the intervention effect) / No information or unpredictable) | Comments |
|  |  |  |  |  |  |  |
|  |  |  |  |  |  |  |

| **(ii) Additional important confounding factors relevant to the setting of this particular study, or identified by the study authors** | | | | | | |
| --- | --- | --- | --- | --- | --- | --- |
| Confounding factor | Measured variable(s) for this factor, if any | Was this variable (or were these variables) controlled for in the analysis?  (Y / N) | If this confounding factor was controlled for, was it measured validly and reliably by this variable (or these variables)?*  (NA / Y / PY / PN / N / NI) | If this confounding factor was not controlled for, is there evidence that controlling for it was unnecessary?**  (NA / Y / PY / PN / N) | OPTIONAL: Is failure to adjust for this confounding factor expected to bias the effect estimate upwards or downwards? (Upward bias (overestimate the intervention effect) / Downward bias (underestimate the intervention effect) / No information or unpredictable) | Comments |
|  |  |  |  |  |  |  |
|  |  |  |  |  |  |  |

* “Validity” refers to whether the confounding variable or variables accurately measure the confounding factor, while “reliability” refers to the precision of the measurement (more measurement error means less reliability).

** In the context of a particular study, variables need not be included in the analysis: (a) ) if they are measured validly and reliably and are not associated with the outcome, conditional on intervention (noting that lack of a statistically significant association is not evidence of a lack of association; (b) if they are measured validly and reliably and are not associated with intervention; (c) if they are measured validly and reliably and adjustment makes no or minimal difference to the estimated effect of the primary parameter; (d) because the confounder was addressed in the study design, for example by restricting to individuals with the same value of the confounder; (e) because a negative control demonstrates that there was unlikely to have been confounding due to this variable or that uncontrolled confounding was likely to be minimal; or (f) because external evidence suggests that controlling for the variable is not necessary in the context of the study being assessed”.

# Risk of bias assessment

Responses underlined in green are potential markers for low risk of bias, and responses in red are potential markers for a risk of bias. Where questions relate only to sign posts to other questions, no formatting is used.

## 1. Bias due to confounding

#### Guidance notes

The questions in this domain focus on the confounding factors that were identified as important in the preliminary evaluation in section E.

We use the term uncontrolled confounding to refer to confounding that was not controlled by the design or analysis of the study – and is therefore likely to bias the estimated effect of intervention. This may arise because (i) confounding factors were not (or could not) be measured; (ii) variables used to measure confounding factors were insufficient to characterize the confounding factor; or (iii) variables that characterize the confounding factor were measured but not included in the analysis.

#### Domain 1, Variant A (only baseline confounding needs to be addressed – if N to C2, or Y to C2 and N to C3)

| **Signalling questions** | **Elaboration** | **Response** |
| --- | --- | --- |
| **1.1 Did the authors control for all the important confounding factors for which this was necessary?** | The important confounding factors are those specified in the *Preliminary consideration of confounding factors*. The preliminary assessment will have determined whether there were important confounding factors that were not controlled for and should have been (because there was no evidence that controlling for the variable was unnecessary). Failure to control for all important confounding factors may lead to bias. The analysis should attempt to control for these confounding factors using an appropriate method, for example using stratification, regression, matching, standardization or inverse probability weighting (control may be for individual variables or for estimated propensity scores).  Answer ‘Y’ or ‘PY’ if all the important confounding factors for which it is was deemed necessary to control (under *Preliminary consideration of confounding factors*) were indeed controlled for appropriately. Also answer ‘Y’ or ‘PY’ in the (very rare) situation that there are no confounding factors and an unadjusted analysis is presented.  Answer ‘WN’ if most of the important confounding factors for which it was deemed necessary to control (under *Preliminary consideration of confounding factors*) were controlled for using appropriate methods, and the any uncontrolled confounding (because not all important confounding factors were controlled for) was not likely to be substantial. This would be the case, for example, if the factors that were not controlled for were likely to be highly correlated with factors that were controlled for.  Answer ‘SN’ if there is at least one important confounding factor that should have been controlled for but was not, and the failure to control for this factor is likely to have a material impact on the estimated effect of intervention. | PY |
| **1.2 If Y/PY/WN to 1.1: Were confounding factors that were controlled for (and for which control was necessary) measured validly and reliably by the variables available in this study?** | Appropriate control of confounding requires that the variables adjusted for are valid and reliable measures of the confounding factors. For some topics, a list of valid and reliable measures of confounding factors will be specified in the review protocol but for others such a list may not be available. Study authors may cite references to support the use of a particular measure. If authors control for confounding variables with no indication of their validity or reliability, then subjectivity of the measure should be evaluated.  In the (very rare) situation that there are no confounding factors and an unadjusted analysis is presented, answer ‘Y’ or ‘PY’. | Y |
| **1.3 If Y/PY/WN to 1.1: Did the authors control for any post-intervention variables that could have been affected by the intervention?** | Controlling for post-intervention variables that are affected by intervention is not appropriate (this is sometimes called ‘over-adjustment’). Controlling for mediating variables estimates the direct effect of intervention and may introduce bias. Controlling for common effects of intervention and outcome (sometimes referred to as ‘colliders’) introduces bias. | N |
| **1.4. Did the use of negative controls, quantitative bias analysis, or other considerations, suggest serious unmeasured confounding?** | Use of a “negative control” – exploration of an alternative analysis in which no association should be observed – can sometimes suggest that the result is subject to uncontrolled confounding, if similar associations are identified for the result being assessed and the negative control.  If the study did not use negative controls and no other considerations suggest uncontrolled confounding, answer ‘N’.  Answer ‘Y’ or ‘PY’ if negative controls indicate that the result being assessed suffers from material bias due to confounding. | N |
| Risk of bias judgement | See algorithm. | Low |
| Optional: What is the predicted direction of bias due to confounding? | If the likely direction of bias can be predicted, it is helpful to state this. A judgement about the predicted direction of bias should take into account all uncontrolled confounding from omitted important confounders and may therefore require judgements about the relative impact of confounding factors operating in different directions.  The effect of confounding is to bias the estimated effect upwards or downwards – to overestimate or to underestimate the intervention effect. If the true intervention effect is above 0 (above 1 for a ratio measure) then an upward bias will also represent a bias away from the null; if the true intervention effect is below 0 (below 1 for a ratio measure) then a downward bias will also represent a bias away from the null. The situation is not always so clear. For example, if the true effect is above 0 then a downward bias may bring the estimate towards the null or beyond it (to a value less than 0). | Upward bias (overestimate the effect) / Downward bias (underestimate the effect) / Unpredictable |

Algorithm for reaching default risk of bias judgement:

#### Domain 1, Variant B (the analysis was based on splitting participants’ follow up time according to intervention received, so both baseline and time-varying confounding need to be addressed – Y to C2 and Y to C3)

| **Signalling questions** | **Elaboration** | **Response** |
| --- | --- | --- |
| **1.1 Did the authors use an analysis method that was appropriate to control for time-varying as well as baseline confounding ?** | Appropriate methods to control for time-varying confounding include those based on inverse probability weighting. Standard regression models that include time-varying confounders may be problematic when the time-varying confounders are affected by prior intervention (this is also known as treatment-confounder feedback). | Y / PY / PN / N / NI |
| **1.2 If Y/PY to 1.1: Did the authors control for all the important baseline and time-varying confounding factors for which this was necessary?** | The important confounding factors are those specified in the *Preliminary consideration of confounding factors*. For studies in which follow up time is split according to intervention received, the confounding factors should include the time-varying confounders (prognostic factors that predict changes to intervention received). The preliminary assessment will have determined whether there were important confounding factors that were not controlled for and should have been (because there was no evidence that controlling for the variable was unnecessary). Failure to control for all important confounding factors may lead to bias.  Answer ‘Y’ or ‘PY’ if all the important confounding factors for which it is was deemed necessary to control (under *Preliminary consideration of confounding factors*) were indeed controlled for. Also answer ‘Y’ or ‘PY’ in the (very rare) situation that there are no confounding factors and an unadjusted analysis is presented.  Answer ‘WN’ if most of the important confounding factors for which it was deemed necessary to control (under *Preliminary consideration of confounding factors*) were controlled for, and the any uncontrolled confounding (because not all important confounding factors were controlled for) was not likely to be substantial. This would be the case, for example, if the factors that were not controlled for were likely to be highly correlated with factors that were controlled for.  Answer ‘SN’ if there is at least one important confounding factor that should have been controlled for but was not, and the failure to control for this factor is likely to have a material impact on the estimated effect of intervention. | NA / Y / PY / WN (no, but uncontrolled confounding was probably not substantial) / SN (no, and uncontrolled confounding was probably substantial) / NI |
| **1.3 If Y/PY/WN to 1.2: Were confounding factors that were controlled for (and for which control was necessary) measured validly and reliably by the variables available in this study?** | As for variant A, question 1.2. | NA / Y / PY / WN (no, but the extent of measurement error in confounding factors was probably not substantial) / SN (no, and the extent of measurement error in confounding factors was probably substantial) / NI |
| **1.4 If N/PN/NI to 1.1: Did the authors control for time-varying factors or other variables measured after the start of intervention?** | This question is asked if an inappropriate analysis method has been used to control for time-varying confounding factors. In such a situation, controlling for (conditioning on) time-varying factors measured after the start of intervention is likely to lead to bias if these are also on the causal pathway from the intervention to the outcome. | Y / PY / PN / N / NI |
| **1.5 Did the use of negative controls, or other considerations, suggest serious unmeasured confounding?** | As for variant A, question 1.4. |  |
| Risk of bias judgement | As for variant A. |  |
| Optional: What is the predicted direction of bias due to confounding? | As for variant A. |  |

Algorithm for reaching default risk of bias judgement:

## 2. Bias in classification of interventions

| **Signalling questions** | **Elaboration** | **Response options** |
| --- | --- | --- |
| *Questions about immortal time bias arising from definition of intervention groups* |  |  |
| **2.1 Did assignment of participants to the intervention group or the comparator group rely on events or measurements that occurred after the start of follow up?** | Using events or measurements that occurred after the start of follow up to assign participants to the intervention or comparator groups may lead to some participants having a period of ‘immortal time’ during which the outcome cannot occur. Examples include (i) interventions defined as receipt of a minimum number of prescriptions, and (ii) intervention defined as eligibility for, followed by subsequent receipt of, a surgical intervention such as heart transplant.  *NB This question is repeated (as question 3.1) in domain 3 (bias in selection of participants into the study). Defining interventions based on events or measurements that occurred after the start of follow up may lead either to misclassification (this domain) or selection bias (domain 3).* | N |
| **2.2 If Y/PY to 2.1: Were participants included in the comparator group until they fulfilled the definition of the intervention (or vice versa)?** | In this situation, participants who would have received the intervention but experienced an outcome event soon after the start of follow up are more likely to be included in the comparator group.  Note that this question does not address situations in which follow-up time is split according to intervention received: in such situations answer ‘N’ or ‘PN’.  Response options ‘WY’ and ‘SY’ are used to distinguish between ‘Serious’ and ‘Critical’ risk of immortal time bias. Answer ‘SY’ if the impact of including participants in the comparator group until they fulfilled the definition of the intervention was likely to be substantial. | NA / SY (yes, and the impact was substantial) / WY (yes, but the impact was not substantial) / PN / N / NI |
| *Questions about differential misclassification* |  |  |
| **2.3 If N/PN to 2.1: Was all information used to classify intervention and comparator groups recorded at or before the time the interventions started?** | Information that was collected after the interventions started may be influenced by the outcome or the risk of the outcome. If information used to classify the intervention and comparator groups came from sources that were recorded at or before the time the interventions started, then differential misclassification (misclassification that depends on the outcome) is less likely.  Answer ‘Y’ or ‘PY’ if, for example, information was collected directly from records of prescriptions.  Answer ‘N’ or ‘PN’ if, for example, participants were asked to recall past interventions. | Y |
| **2.4 Was classification of intervention status influenced by knowledge of the outcome or risk of the outcome?** | Even if intervention status is recorded at the start of intervention, it may be possible for outcome events to affect its availability for the purposes of classifying outcomes of the non-randomized study. For example, if records of receipt of an intervention are destroyed if a participant dies (leading to misclassification of that participant into the comparator group) then analyses investigating the effect of the intervention on mortality will be biased due to non-differential misclassification depending on the outcome.  Response options ‘WY’ and ‘SY’ are used to distinguish between ‘Serious’ and ‘Critical’ risk of bias arising from classification of intervention status. Answer ‘SY’ if the impact of knowledge of the outcome or risk of bias outcome was likely to be substantial. | N |
| *Question about non-differential misclassification* |  |  |
| **2.5 If N/PN to 2.1 and WY/N/PN/NI 2.4: Was intervention status classified correctly for all, or nearly all, participants?** | This question relates to non-differential misclassification (that is, unrelated to the subsequent outcome) of intervention status. For example, intervention status may be misclassified for some participants if receipt of (or assignment to) the intervention is not recorded in the information source used to classify intervention status. Similarly, some participants may receive (or be assigned to) the intervention without it being recorded. Misclassification errors of this nature usually bias the result towards the null.  Questions about non-differential misclassification are asked only if no serious problems have been identified in relation to immortal time bias (questions 2.1 to 2.3) or differential misclassification (questions 2.4 to 2.6).  Criteria for considering individuals to have received each intervention should be clear and explicit, covering issues such as type, setting, dose, frequency, intensity and timing of intervention. A pre-requisite for correct classification of interventions is that the interventions are well defined. Ambiguity in the definition may lead to misclassification of participants, and so is likely to lead to a response of ‘No’ or ‘Probably no’ to this question.  It may be helpful to think separately about (i) whether all people who were assigned to (or who received) the intervention were correctly classified, and (ii) whether all people who were assigned to (or who received) the comparator were correctly classified.  “Nearly all” should be interpreted as “enough to be confident of the findings”, and a suitable proportion will depend on the context. In many situations, correct classification for 95% of the participants may be sufficient.  Response options ‘WN’ and ‘SN’ are used to distinguish between ‘Moderate’ and ‘Serious’ risk of bias when there is no risk of immortal time bias (from question 2.1) or differential misclassification (from question 2.4). | Y |
| Risk of bias judgement | See algorithm. | Low |
| Optional: What is the predicted direction of bias in classification of interventions? | If the likely direction of bias can be predicted, it is helpful to state this. The direction might be characterized as being in favour of the intervention, as being in favour of the comparator, or as towards (or away from) the null. Non-differential misclassification will often bias results towards the null. | Favours intervention / Favours comparator / Towards null /Away from null / Unpredictable |

Algorithm for reaching default risk of bias judgement:

## 3. Bias in selection of participants into the study (or into the analysis)

In the target trial, start of follow up is the time at which participants meet eligibility criteria and are assigned to interventions. In answering the signalling questions for this domain, consider what is the start of follow up in the study under consideration, for both the intervention and comparison groups.

| **Signalling questions** | **Elaboration** | **Response options** |
| --- | --- | --- |
| *A. Questions about immortal time bias arising from definition of intervention groups* |  |  |
| **3.1 (=2.1) Did assignment of participants to the intervention group or the comparator group rely on events or measurements that occurred after the start of follow up?** | Using events or measurements that occurred after the start of follow up to assign participants to the intervention or comparator groups may lead to some participants having a period of ‘immortal time’ during which the outcome cannot occur. Examples include (i) interventions defined as receipt of a minimum number of prescriptions, and (ii) intervention defined as eligibility for, followed by subsequent receipt of, a surgical intervention such as heart transplant.  *NB This question has previously been answered (as question 2.1) in domain 2 (bias in classification of interventions). Defining interventions based on events or measurements that occurred after the start of follow up may lead either to misclassification (domain 2) or selection bias (this domain).* | N |
| **3.2 If Y/PY to 3.1: Were participants excluded after the start of follow-up because they did not meet the definition of either the intervention or the comparator?** | In this situation, participants who would have received the intervention but experienced an outcome event soon after the start of follow up are more likely to be excluded, leading to immortal time bias. | NA / Y / PY / PN / N / NI |
| *B. Questions about prevalent user bias* |  |  |
| **3.3 Were start of follow up and start of intervention the same for most participants?** | If participants are not followed from the start of the intervention then a period of follow up has been excluded, and individuals who experienced the outcome soon after intervention will be missing from analyses. This problem may occur when prevalent, rather than new (incident), users of the intervention are included in analyses.  If the comparison group is “no intervention” then follow up can start at any point at which participants remain eligible to start the intervention. | Y |
| **3.4 If N/PN to 3.3: Is the effect of intervention expected to be constant over the time period studied?** | Consider the effect of intervention (for example, rate ratio) that is estimated by the result being assessed. If the effect varies over time the estimate based on follow up from the start of intervention will differ from the effect restricted to later follow up | NA / Y / PY / PN / N / NI |
| *C. Questions about other types of selection bias* |  |  |
| **3.5 Was selection of participants into the study (or into the analysis) based on participant characteristics observed after the start of intervention (additional to the situations addressed in 3.1 and 3.3)?** | Answer ‘Y’ or ‘PY’ to this question if selection into the study was based on participant characteristics observed *after* the start of intervention, for reasons additional to those addressed in parts A and B of this domain. Answer ‘N’ or ‘PN’ if selection was based only on characteristics observed *before* the start of intervention. Such selection can be addressed by controlling for imbalances between intervention and comparator groups in baseline characteristics that are prognostic for the outcome (baseline confounding).  This domain *does not* address exclusion because of missing data (incomplete information on a variable measured in some participants). | N |
| **3.6 If Y/PY to 3.5: Were the post-intervention variables that influenced selection likely to be associated with intervention?** | Selection bias occurs when selection is related to an effect of either intervention or a cause of intervention **and** an effect of either the outcome or a cause of the outcome. Therefore, the result is at risk of selection bias if selection into the study is related to both the intervention and the outcome. | NA / Y / PY / PN / N / NI |
| **3.7 If Y/PY to 3.6: Were the post-intervention variables that influenced selection likely to be influenced by the outcome or a cause of the outcome?** |  | NA / Y / PY / PN / N / NI |
| *D. Questions about analysis, sensitivity analyses and severity of the problem* |  |  |
| **3.8 If Y/PY to 3.2, N/PN 3.4 or Y/PY to 3.7: Is it likely that the analysis corrected for all of the potential selection biases identified in 3.1-3.2, 3.3-3.4 or 3.5-3.7 above?** | It is in principle possible to correct for selection biases, for example by: (1) using inverse probability weights to create a pseudo-population in which the selection bias has been removed; (2) modelling the distributions of the missing follow up times and outcome events in prevalent users of intervention and including them using missing data methodology; or (3) using the three-step ‘clone, censor weight’ approach proposed by Hernán to avoid immortal time bias. Answer ‘Y‘ or ‘PY‘ if appropriate methods were used and the assumptions required for there validity were likely to be justified. Otherwise, the answer to this question will usually be ‘N’ or ‘PN’. | NA / Y / PY / PN / N / NI |
| **3.9 If N/PN to 3.8: Did sensitivity analyses demonstrate that the likely impact of the potential selection biases identified in 3.1-3.2, 3.3-3.4 or 3.5-3.7 above was minimal?** |  | NA / Y / PY / PN / N / NI |
| **3.10 If N/PN to 3.9: Were potential selection biases identified in 3.1-3.2, 3.3-3.4 or 3.5-3.7 above sufficiently severe that the result should not be included in a quantitative synthesis?** | This question is used to distinguish between ‘Serious’ and ‘Critical’ risk of selection bias. Answer ‘N’, ‘PN’ or ‘NI’ unless there is clear evidence that the selection biases identified were severe. | NA / Y / PY / PN / N / NI |
| Risk of bias judgement | See algorithm. | Low |
| Optional: What is the predicted direction of bias in selection of participants into the study? | If the likely direction of bias can be predicted, it is helpful to state this. The direction might be characterized as being in favour of the intervention, as being in favour of the comparator, or as towards (or away from) the null. | Favours intervention / Favours comparator / Towards null /Away from null / Unpredictable |

Algorithm for reaching default risk of bias judgement

## 4. Bias due to deviations from intended interventions

#### Domain 4, Variant A: Effect of assignment to intervention

| **Signalling questions** | **Elaboration** | **Response options** |
| --- | --- | --- |
| **4.1 Was the study undertaken in an experimental context?** | When interest focusses on the effect of assignment to intervention, bias (in this domain) only arises if there are deviations that arose because the study was done in an experimental context. Although most non-randomized studies are observational rather than experimental, examples of experimental contexts are (i) controlled trials that use methods other than randomization to allocate interventions to participants; and (ii) studies in which participants (or other units such as schools, clinics or administrative bodies) self-select to receive an intervention for the purposes of a comparison of their outcomes with those from participants (or other units) who do not.  The answer ‘N’ or ‘PN’ will be appropriate for most observational studies of interventions.  Answer ‘Y’ or ‘PY’ only if participants were allocated to interventions as part of the study. | N |
| **4.2. If Y/PY to 4.1: Did participants deviate from the intended intervention as a result of the processes of recruiting and engaging them in the study?** | This question refers to the possibility that study recruitment and engagement activities lead study participants to deviate from the intended intervention in a way that would not happen outside the experimental context. For example, the process of securing informed consent may lead participants subsequently assigned to the comparator group to feel unlucky and therefore seek the intervention, or other interventions that improve their prognosis.  Answer ‘Y’ or ‘PY‘ **only in the rare situations when** there is evidence, or strong reason to believe, that the processes of recruiting and engaging with study participants led to non-adherence to the intended intervention strategies.  The answer ‘NI’ may be appropriate, and maps to low risk of bias for this domain.  The answer ‘N’ or ‘PN’ will usually be appropriate, including when there was non-adherence to assigned intervention that is consistent with what could occur outside the experimental context.  Answer ‘N’ or ‘PN’ for non-adherence to intervention that is consistent with the study protocol, for example cessation of a drug intervention because of acute toxicity. | NA / Y / PY / PN / N / NI |
| **4.3. If Y/PY to 4.1: Did study personnel consciously or unconsciously undermine implementation of the intended interventions?** | This question refers to situations in which study personnel (care givers or people delivering the interventions) undermine implementation of the intervention in ways that would not happen outside the experimental context of the study. “Study personnel” includes anyone involved in implementing an intended intervention who is aware of the ongoing study and aware of the comparison(s) being made across intervention groups.  Changes to intervention that are consistent with the study protocol, for example use of additional interventions whose aim is to treat adverse effects of one of the interventions, are not deviations from intended intervention and **should not** influence the answer to this question.  Answer ‘Y’ or ‘PY’ **only** if there is evidence, or strong reason to believe, that actions (or inactions) of study personnel, driven by conscious or unconscious biases, led to failure to implement the specified intervention strategies. For example, in an open-label study comparing minimally invasive versus open surgery for oesophageal cancer the protocol specified that one-lung ventilation should be used in both groups (see Example 3 in Box 1). However, one-lung mechanical ventilation is thought to increase respiratory complications including RTIs, and surgeons usually used two-lung ventilation in the minimally invasive group.  The answer ‘NI’ may be appropriate, and maps to low risk of bias for this domain.  Answer ‘N’ or ‘PN’ if failure to implement the intervention was consistent with what could occur outside the experimental context. | NA / Y / PY / PN / N / NI |
| **4.4. If Y/PY/NI to 4.2 or 4.3: Were these deviations from intended intervention likely to have affected the outcome?** | Deviations from intended interventions that do not reflect usual practice (i.e. were due to the experimental context) will be important if they affect the outcome, but not otherwise. | NA / Y / PY / PN / N / NI |
| **4.5. Was an appropriate analysis used to estimate the effect of assignment to intervention?** | An analysis to estimate the effect of assignment to intervention should include all individuals regardless of whether they deviated from the assigned intervention strategy after the start of follow-up. If the analysis excluded participants or follow-up because of post-baseline deviations from the assigned intervention, the result is at risk of bias. The issue is analogous to the use of an intention-to-treat (ITT) analysis in a randomized trial.  Response options ‘WY’ and ‘SY’ are used to distinguish between different risks of bias. Answer 'SN' if the extent of deviations from the intended intervention was sufficient that there could have been a substantial impact on the result. It is not possible to specify a precise rule: there may be potential for substantial impact even if fewer than 5% of participants were analysed in the wrong group or excluded, if the outcome is rare or if exclusions are strongly related to prognostic factors. | Y |
| Risk of bias judgement | See algorithm. | Low |
| Optional: What is the predicted direction of bias in classification of interventions? | If the likely direction of bias can be predicted, it is helpful to state this. The direction might be characterized as being in favour of the intervention, as being in favour of the comparator, or as towards (or away from) the null. | Favours intervention / Favours comparator / Towards null /Away from null / Unpredictable |

Algorithm for reaching default risk of bias judgement (effect of assignment to intervention)

#### Domain 4, Variant B: Per-protocol effect (effect of adhering to intervention)

In answering the signalling questions below, consider the intervention strategies and protocol deviations specified in the preliminary considerations.

| **Signalling questions** | **Elaboration** | **Response options** |
| --- | --- | --- |
| **4.1 Did all or nearly all participants adhere to their assigned intervention strategy?** | Answer ‘Y’ or ‘PY’ if the changes to intervention or comparator strategies considered to be protocol deviations (see preliminary considerations) did not occur for most participants. | Y |
| **4.2. If N/PN/NI to 4.1: Were the protocol deviations likely to have affected the outcome?** | Protocol deviations will be important if they affect the outcome, but not otherwise. | NA / Y / PY / PN / N / NI |
| **4.3 If Y/PY to 4.2: Was an appropriate analysis used to estimate the specified per-protocol effect, accounting for the specified protocol deviations?** | It is possible to conduct appropriate analyses to estimate per-protocol effects, for example using inverse probability weighting to adjust for censoring of participants who cease adherence to their assigned intervention. These methods depend on strong assumptions, which should be appropriate and justified if the answer to this question is ‘Y’ or ‘PY’. It is possible that a paper reports such an analysis without reporting information on the protocol deviations that are accounted for, but it would be hard to judge such an analysis to be appropriate in the absence of such information. | NA / Y / PY / PN / N / NI |
| Risk of bias judgement | See algorithm. | Low |
| Optional: What is the predicted direction of bias in classification of interventions? | If the likely direction of bias can be predicted, it is helpful to state this. The direction might be characterized as being in favour of the intervention, as being in favour of the comparator, or as towards (or away from) the null. | Favours intervention / Favours comparator / Towards null /Away from null / Unpredictable |

Algorithm for reaching default risk of bias judgement (effect of adhering to intervention)

## 5. Bias due to missing data

#### Guidance notes

Missing outcome data may arise, among other reasons, through attrition (loss to follow up), missed appointments and incomplete data collection. Additionally, in non-randomized studies data may be missing for characteristics including interventions received and confounders.

A general rule for consideration of bias due to missing data is that we they should consider biases introduced by the missing data, compared with the effect estimate from an analysis in which all the data we intended to collect were available. Unfortunately, a single threshold for an acceptable proportion of missing data cannot meaningfully be defined. For example, a result based on 95% complete outcome data might be biased if the outcome was rare and if reasons for missing outcome data were strongly related to intervention group. Therefore, the potential for bias due to missing data should be assessed unless complete data on intervention status, the outcome and confounding variables were available all, or nearly all, participants.

Considerations of bias due to missing data depend on how the analysis accounted for the missing data. Different signalling questions should be answered depending on three types of analysis. The first is that a **complete case analysis**, restricted to participants with complete data on all the intervention, outcome and confounding variables, was performed. In this situation, an important consideration is whether missingness of individual participants from the analysis is related to the true value of the outcome for those participants. The second is that missing data were **imputed**, which means that estimated or assumed values were assigned to participants with missing data. Imputed data should not lead to bias if the data are ‘missing at random’ (see the elaboration for signalling question 5.8) and an appropriate imputation method is applied. Other types of analysis are addressed by a separate, general, signalling question. The final signalling question asks whether sensitivity analyses were performed that demonstrated that the impact of missing data is minimal.

| **Signalling questions** | **Elaboration** | **Response options** |
| --- | --- | --- |
| **5.1 Were complete data on intervention status available for all, or nearly all, participants?** | “Nearly all” should be interpreted as that the number of participants excluded from the analysis due to missing data is so small that they could have made no important difference to the estimated effect of the intervention.  Only answer ‘NI’ if the study report provides no information about the extent of missing data. This situation will usually lead to a judgement that there is a high risk of bias due to missing data.  Note that this question refers to data actually recorded in the study. Imputed data (see question 5.7) should be regarded as missing data in the context of this question. | Y |
| **5.2 Were complete data on the outcome available for all, or nearly all, participants?** | “Nearly all” should be interpreted as that the number of participants excluded from the analysis due to missing data is so small that they could have made no important difference to the estimated effect of the intervention.  For continuous outcomes, complete data for 95% (or possibly 90%) of the participants would often be sufficient. For dichotomous outcomes, the proportion required is directly linked to the risk of the outcome event. If the observed number of outcome events is much greater than the number of participants with missing data, the bias would necessarily be small.  Only answer ‘NI’ if the study report provides no information about the extent of missing data. This situation will usually lead to a judgement that there is a high risk of bias due to missing data.  Note that this question refers to data actually recorded in the study. Imputed data should be regarded as missing data in the context of this question. | Y |
| **5.3 Were complete data on important confounding variables available for all, or nearly all, participants?** | “Nearly all” should be interpreted as that the number of participants excluded from the analysis due to missing data is so small that they could have made no important difference to the estimated effect of the intervention.  Only answer ‘NI’ if the study report provides no information about the extent of missing data. This situation will usually lead to a judgement that there is a high risk of bias due to missing data.  Note that this question refers to data actually recorded in the study. Imputed data should be regarded as missing data in the context of this question. | Y |
| **5.4 If N/PN/NI to 5.1, 5.2 or 5.3: Is the result based on a complete case analysis?** | How risk of bias is assessed depends on whether a complete case analysis has been done. A complete case analysis is one that is restricted to participants with complete data on all the intervention, outcome and confounding variables. | NA / Y / PY / PN / N / NI |
| **5.5 If Y/PY/NI to 5.4: Was exclusion from the analysis because of missing data (in intervention, confounders or the outcome) likely to be related to the true value of the outcome?** | This question aims to identify situations in which a “complete case” analysis will be at risk of bias due to missing data. A complete case analysis is one that includes all participants who provide full data for the variables involved in the analysis.  A complete case analysis may be biased if missingness (in the intervention, outcome or confounders) is related to the outcome. For example, if it is likely that participants with underlying health problems missed a visit at which baseline intervention status or confounders should have been measured, their consequent exclusion from the analysis may be related to their eventual outcome.  Four reasons for answering ‘Y’ or ‘PY’ are:   (1) There are differences between intervention groups or confounder groups/levels in the proportions of participants excluded from the analysis due to missing outcome data. For time-to-event-data, the analogue is that rates of censoring (loss to follow-up) depend on the intervention group. (2) There are differences between outcome groups/levels in the proportions of participants excluded from the analysis due to missing intervention/confounder data. (3) Reported reasons for missing outcome data provide evidence that missingness in the outcome depends on the true outcome or a cause of the outcome; (4) It is reasonable to assume that the circumstances of the study make it likely that missingness in the outcome depends on its true value. For example, if the outcome is severe depression, it may be likely that participants experiencing the outcome miss appointments at which it would have been recorded.   Answer ‘N’ or ‘PN’ if missing data, loss to follow up or withdrawal occurred for documented reasons that are unrelated to the outcome, in which case the risk of bias due to missing data will be low. | NA / Y / PY / PN / N / NI |
| **5.6 If Y/PY/NI to 5.5:** **Is the relationship between the outcome and missingness likely to be explained by the variables in the analysis model?** | If all the variables that plausibly explain the relationship between the outcome and missingness (in intervention, confounders or the outcome) are included in the complete case analysis, then the risk of bias will be low. For example, in a regression of blood pressure at age 55 (outcome) on reducing salt intake (intervention) that adjusts for the confounders sex, education level and blood pressure measured at age 25, if women were more likely to have blood pressure measured at age 25 and if sex is the only variable plausibly related to missingness, this would not cause bias because sex is adjusted for in the analysis model. Hence blood pressure at age 55 is not related to missingness in blood pressure at age 25, once sex is adjusted for.  Technical note: If a mediator (a variable on the causal pathway from intervention to outcome) affects missingness, than adjusting for this variable would be appropriate to avoid bias due to missing data in a complete case analysis, but would change the intervention effect being estimated. In the presence of such variables, multiple imputation (see question 5.7) should be used to address bias due to missing data. Adjusting for a mediator will increase the risk of bias due to confounding (domain 1). | NA / Y / PY / WN (No, but not leading to substantial bias) / SN (No, and bias is likely to be substantial) / NI |
| **5.7 If N/PN to 5.4: Was the analysis based on imputing missing values?** | Imputing missing values is the process of assigning estimated or assumed values to them for use in the main analysis.  Answer ‘Y’ or ‘PY’ if the analysis was based on either single or multiple imputation. | NA / Y / PY / PN / NI |
| **5.8 If Y/PY to 5.7: Is it reasonable to assume that data were ‘missing at random’ (MAR) or ‘missing completely at random’ (MCAR)?** | In their book *Statistical analysis with missing data* (Wiley 2002), Little and Rubin proposed commonly-used categorizations of missing data. These were summarized by Sterne et al (BMJ 2009; **338**: b2393) as follows:  *Missing completely at random* (MCAR): There are no systematic differences between the missing values and the observed values. For example, blood pressure measurements may be missing because of breakdown of an automatic sphygmomanometer.  *Missing at random* (MAR): any systematic difference between the missing values and the observed values can be explained by differences in observed data. For example, missing blood pressure measurements may be lower than measured blood pressures but only because younger people may be more likely to have missing blood pressure measurements.  *Missing not at random* (MNAR): Even after the observed data are taken into account, systematic differences remain between the missing values and the observed values. For example, people with high blood pressure may be more likely to miss clinic appointments because they have headaches.  Analyses based on multiple imputation can avoid bias due to missing data provided that the incomplete variables for which data are imputed are MAR or MCAR, but not if data are MNAR.  Answer ‘N’ or ‘PN’ if there is a reason to believe that data are missing not at random (MNAR). Otherwise, answer ‘Y’ or ‘PY’. | NA / Y / PY / PN / N / NI |
| **5.9 If Y/PY to 5.8: Was imputation performed appropriately?** | Answer ‘SN’ or ‘WN’ if simple imputation methods such as last observation carried forward or imputing a mean value are used. The amount of bias likely to be introduced by this will depend on the proportion of participants with missing data.  Answer ‘Y’ or ‘PY’ if multiple imputation was used and (i) all the predictors of missingness in any variable were included in the imputation models; and (ii) all the variables in the model used for the main analysis were included in the imputation models. | NA / Y / PY / WN (no, but not leading to substantial bias) / SN (no, such that bias would not be substantially reduced) / NI |
| **5.10 If N/PN/NI to 5.7: Was an appropriate alternative method used to correct for bias due to missing data?** | This signalling question covers situations in which the analysis was neither a complete case analysis nor based on imputing missing values. Examples of such analyses include inverse probability weighting and full information maximum likelihood. If weighting is used, its validity depends on the weighting model being correctly specified (see Seaman and White, Stat Methods Med Res 2013; 22: 278-95).  In these situations, the ROBINS-I assessor (possibly in conjunction with a statistician knowledgeable about missing data methods) should attempt to determine whether the analysis was appropriate to correct for any biases. | NA / Y / PY / WN (no, but not leading to substantial bias) / SN (no, such that bias would not be substantially reduced) / NI |
| **5.11 If PN/N/NI to 5.1, 5.2 or 5.3 AND (Y/PY/NI to 5.5 OR (Y/PY to 5.8 AND WN/SN/NI to 5.9) OR WN/SN/NI to 5.10): Is there evidence that the result was not biased by missing data?** | Evidence that the result was not biased by missing data may come from:   (1) analysis methods that would not be biased under plausible relationships between the missing values and the likelihood that data are missing; or (2) sensitivity analyses showing that results are little changed under a range of plausible assumptions about the missing values.   Note that multiple imputation based only on outcome, intervention and confounder information should not be assumed to correct for bias due to missing data, so similarity between results with and without such imputation should not be taken as reassurance when answering this question. Similarly, a weighted analysis should not be assumed to correct for bias due to missing data without further consideration of (1) and (2) above, or sensitivity analyses. | NA / Y / PY / PN / N |
| Risk of bias judgement | See algorithm. | Low |
| Optional: What is the predicted direction of bias due to missing data? | If the likely direction of bias can be predicted, it is helpful to state this. The direction might be characterized as being in favour of the intervention, as being in favour of the comparator, or as towards (or away from) the null. | Favours intervention / Favours comparator / Towards null /Away from null / Unpredictable |

Algorithm for reaching default risk of bias judgement:

## 6. Bias in measurement of the outcome

#### Guidance notes

Bias may be introduced if outcomes are misclassified or measured with error. Misclassification or measurement error of outcomes may be non-differential or differential.

**Non-differential measurement error** is unrelated to the intervention received. It can be systematic (for example when measurement of blood pressure is consistently 5 units too high in every participant) – in which case it will not affect precision or cause bias; or it can be random (for example when measurement of blood pressure is sometimes too high and sometimes too low in a manner that does not depend on the intervention or the outcome) – in which case it will affect precision without causing bias.

**Differential measurement error** is measurement error related to intervention received. It will bias the intervention-outcome relationship. This is often referred to as detection bias. Examples of situations in which detection bias can arise are (i) if outcome assessors are aware of intervention received (particularly when the outcome is subjective); (ii) different methods (or intensities of observation) are used to assess outcomes of participants receiving different interventions; and (iii) measurement errors are related to intervention received (or to a confounder of the intervention-outcome relationship).

Blinding of outcome assessors aims to prevent systematic differences in measurements according to intervention received. However, blinding is frequently not possible or not performed for practical reasons.

| **Signalling questions** | **Elaboration** | **Response options** |
| --- | --- | --- |
| **6.1 Could measurement or ascertainment of the outcome have differed between intervention groups?** | Comparable methods of outcome measurement (data collection) involve the same measurement methods and thresholds, used at comparable time points. Differences between intervention groups may arise because of ‘diagnostic detection bias’ in the context of passive collection of outcome data, or if an intervention involves additional visits to a healthcare provider, leading to additional opportunities for outcome events to be identified. | N |
| **6.2 Were outcome assessors aware of the intervention received by study participants?** | Answer ‘N’ if outcome assessors were blinded to intervention status. In other situations, outcome assessors may be unaware of the interventions being received by participants despite there being no active blinding by the study investigators; the answer this question would then also be ‘No’. In studies where participants report their outcomes themselves, for example in a questionnaire, the outcome assessor is the study participant. In an observational study, the answer to this question will usually be ‘Y’ when the participants report their outcomes themselves. | Y |
| **6.3 If Y/PY/NI to 6.2: Could assessment of the outcome have been influenced by knowledge of the intervention received?** | Knowledge of the assigned intervention could influence participant-reported outcomes (such as level of pain), observer-reported outcomes involving some judgement, and intervention provider decision outcomes.  Knowledge of the assigned intervention is unlikely to influence observer-reported outcomes that do not involve judgement, for example all-cause mortality or laboratory measures of levels of substances in the blood.  The response options distinguish between situations in which (i) knowledge of intervention status could have influenced outcome assessment but there is no reason to believe that it did from those in which (ii) knowledge of intervention status was likely to influence outcome assessment. When there are strong levels of belief in or preference for either beneficial or harmful effects of the intervention, it is more likely that the outcome was influenced by knowledge of the intervention received. Examples justifying the answer ‘SY’ may include patient-reported symptoms in studies of homeopathy, or assessments of recovery of function by a physiotherapist. | N |
| Risk of bias judgement | See algorithm. | Low |
| Optional: What is the predicted direction of bias in measurement of outcomes? | If the likely direction of bias can be predicted, it is helpful to state this. The direction might be characterized as being in favour of the intervention, as being in favour of the comparator, or as towards (or away from) the null. | Favours intervention / Favours comparator / Towards null /Away from null / Unpredictable |

Algorithm for reaching default risk of bias judgement:

## 7. Bias in selection of the reported result

#### Guidance notes

Selective reporting can arise for both harms and benefits of an intervention, although the motivations (and direction of bias) underlying selective reporting of effect estimates for harms and benefits may differ. Selective reporting may arise, for example, from a desire for findings to be newsworthy (or sufficiently noteworthy to merit publication), or from commercial considerations, or from a desire to demonstrate that there is not evidence of a harmful effect of an intervention.

**Selective outcome reporting** occurs when the effect estimate for an outcome measurement was selected from among analyses of multiple outcome measurements for the outcome domain. Examples include: use of multiple measurement instruments (e.g. pain scales) and reporting only the most favourable result; reporting only the most favourable subscale (or a subset of subscales) for an instrument when measurements for other subscales were available; reporting only one or a subset of time points for which the outcome was measured.

**Selective analysis reporting** occurs when results are selected from effects estimated in multiple ways: e.g. carrying out analyses of both change scores and post-intervention scores adjusted for baseline; multiple analyses of a particular measurement with and without transformation; multiple analyses of a particular outcome with and without adjustment for potential confounders (or with adjustment for different sets of potential confounders); multiple analyses of a particular outcome with and without, or with different, methods to take account of missing data; a continuously scaled outcome converted to categorical data with different cut-points; multiple composite outcomes analysed for one outcome domain, but results were reported only for one (or a subset) of the composite outcomes. (Reporting an effect estimate for an unusual composite outcome might be evidence of such selective reporting.)

**Selection of a subgroup from a larger cohort**: The cohort for analysis may have been selected from a larger cohort for which data were available on the basis of a more interesting finding. Subgroups defined in unusual ways (e.g. an unusual classification of subgroups by dose or dose frequency) may provide evidence of such selective reporting.

The best evidence that results were not selectively reported is available if a pre-specified, publicly available analysis plan is available (e.g. from a link in a publication or from an online platform) and is in line with the reported results. Protocols for non-randomized studies are increasingly being registered, although there is inconsistency across platforms (Malmsiø et al, 2022). An analysis plan that is sufficiently detailed to permit full assessment of selective reporting may seldom be available for observational studies. In the absence of a protocol or analysis plan, clues can sometimes be gained by comparing Methods sections with Results sections.

Malmsiø D, Frost A, Hróbjartsson A. A scoping review finds that guides to authors of protocols for observational epidemiological studies varied highly in format and content. J Clin Epidemiol. 2022 Dec 20;154:156-166. doi: 10.1016/j.jclinepi.2022.12.012.

| **Signalling questions** | **Elaboration** | **Response options** |
| --- | --- | --- |
| **7.1 Was the result reported in accordance with an available, pre-determined analysis plan?** | If the researchers’ pre-specified intentions are available in sufficient detail, then planned outcome measurements and analyses can be compared with those presented in the published report(s). To avoid the possibility of selection of the reported result, finalization of the analysis intentions must precede availability of unblinded outcome data to the study authors.  Such analysis plans are rarely publicly available for non-randomized studies, so it is unlikely that a study will be assessed as at low risk of bias for this domain. | N |
| **Is the numerical result being assessed likely to have been selected, on the basis of the results, from...** |  |  |
| **7.2 ... multiple outcome *measurements* (e.g. scales, definitions, time points) within the outcome domain?** | A particular outcome domain (i.e. a true state or endpoint of interest) may be **measured** in multiple ways. For example, the domain pain may be measured using multiple scales (e.g. a visual analogue scale and the McGill Pain Questionnaire), each at multiple time points (e.g. 3, 6 and 12 weeks post-treatment). If multiple measurements were made, but only one or a subset is reported on the basis of the results (e.g. statistical significance), there is a high risk of bias in the fully reported result.  Answer ‘Y’ or ‘PY’ if**:**  There is clear evidence (usually through examination of a study protocol or statistical analysis plan) that a domain was measured in multiple ways, but data for only one or a subset of measures is fully reported (without justification), and the fully reported result is likely to have been selected on the basis of the results. Selection on the basis of the results arises from a desire for findings to be newsworthy, sufficiently noteworthy to merit publication, or to confirm a prior hypothesis. For example, analysts who have a preconception, or vested interest in showing, that an intervention is beneficial may be inclined to report outcome measurements selectively that are favourable to the intervention.  Answer ‘N’ or ‘PN’ if:  There is clear evidence (usually through examination of a study protocol or statistical analysis plan) that all reported results for the outcome domain correspond to all intended outcome measurements.  or  There is only one possible way in which the outcome domain can be measured (hence there is no opportunity to select from multiple measures).  or  Outcome measurements are inconsistent across different reports on the same study, but the analysts have provided the reason for the inconsistency and it is not related to the nature of the results.  Answer ‘NI’ if:  Analysis intentions are not available, or the analysis intentions are not reported in sufficient detail to enable an assessment, and there is more than one way in which the outcome domain could have been measured. | N |
| **7.3 ... multiple *analyses* of the data?** | Because of the limitations of using data from non-randomized studies for analyses of effectiveness (need to control confounding, substantial missing data, etc), analysts may implement different analytic methods to address these limitations. Examples include unadjusted and adjusted models; use of final value vs change from baseline vs analysis of covariance; exploration of different ways of defining intervention and control groups; transformations of variables; a continuously scaled outcome converted to categorical data with different cut-points; different sets of covariates for adjustment; and different strategies for dealing with missing data. Application of multiple methods generates multiple effect estimates for a specific outcome domain. If multiple estimates are generated but only one or a subset is reported, there is a risk of selective reporting on the basis of results (e.g. statistical significance).  Answer ‘Y’ or ‘PY’ if :  There is clear evidence (usually through examination of a study protocol or statistical analysis plan) that a domain was analysed in multiple ways, but data for only one or a subset of analyses is fully reported (without justification), and the fully reported result is likely to have been selected on the basis of the results. Selection on the basis of the results arises from a desire for findings to be newsworthy, sufficiently noteworthy to merit publication, or to confirm a prior hypothesis. For example, analysts who have a preconception or vested interest in showing that an intervention is beneficial may be inclined to report analyses selectively that are favourable to the intervention.  Answer ‘N’ or ‘PN’ if :  There is clear evidence (usually through examination of a study protocol or statistical analysis plan) that all reported results for the outcome domain correspond to all intended analyses.  or  There is only one possible way in which the outcome domain can be analysed (hence there is no opportunity to select from multiple analyses).  or  Analyses are inconsistent across different reports on the same study, but the analysts have provided the reason for the inconsistency and it is not related to the nature of the results.  Answer ‘NI’ if :  Analysis intentions are not available, or the analysis intentions are not reported in sufficient detail to enable an assessment, and there is more than one way in which the outcome domain could have been analysed. | PY |
| **7.4 ... multiple *subgroups*?** | Particularly with large cohorts often available from routinely collected data sources, it is possible to generate multiple effect estimates for different subgroups or simply to omit varying proportions of the original cohort. If multiple estimates are generated but only one or a subset is reported, there is a risk of selective reporting on the basis of results (e.g. statistical significance).  Answer ‘Y’ or ‘PY’ if :  There is clear evidence (usually through examination of a study protocol or statistical analysis plan) that different subgroups were analysed, but data for only one or a subset of analyses is fully reported (without justification), and the fully reported result is likely to have been selected on the basis of the results. Selection on the basis of the results arises from a desire for findings to be newsworthy, sufficiently noteworthy to merit publication, or to confirm a prior hypothesis. For example, analysts who have a preconception or vested interest in showing that an intervention is beneficial may be inclined to report results selectively for subgroups that are favourable to the intervention.  Answer ‘N’ or ‘PN’ if :  There is clear evidence (usually through examination of a study protocol or statistical analysis plan that was date-stamped before the analyst had access to the collected data) that all reported results for the subgroups correspond to all intended analyses.  or  Analyses are inconsistent across different reports on the same study, but the analysts have provided the reason for the inconsistency and it is not related to the nature of the results.  Answer ‘NI’ if :  Analysis intentions are not available, or the analysis intentions are not reported in sufficient detail to enable an assessment, and there is more than one way in which subgroups could have been analysed. | N |
| Risk of bias judgement | See algorithm. | Serious risk of bias |
| Optional: What is the predicted direction of bias in selection of the reported result? | If the likely direction of bias can be predicted, it is helpful to state this. The direction might be characterized as being in favour of the intervention, as being in favour of the comparator, or as towards (or away from) the null. | Favours intervention / Favours comparator / Towards null /Away from null / Unpredictable |

Algorithm for reaching default risk of bias judgement:

.

## Overall risk of bias

#### Guidance notes

ROBINS-I defaults to setting the overall risk of bias for a result to be equal to the risk-of-bias judgement for the domain with the greatest risk of bias. For example, if the ‘worst’ judgement across domains is of serious risk of bias, then the result would be judged as at serious risk of bias overall. However, the user may override this to judge the result to be at greater risk of bias if there are problems in several domains. For example, if several domains are assessed to be at serious risk of bias, and it is considered that these problems are likely to be compounded, then it may be reasonable to judge the result to be at critical risk of bias overall.

Predicting the direction of bias overall may be difficult. Risk-of-bias judgements for the individual domains might be used to inform the influence of that domain to the likely direction of bias overall.

| Overall risk of bias | See algorithm. | Serious risk |
| --- | --- | --- |
| What is the predicted direction of bias? | If the likely direction of bias can be predicted, it is helpful to state this. The direction might be characterized as being in favour of the intervention, as being in favour of the comparator, or as towards (or away from) the null. Alternatively, if there direction is driven by bias due to confounding, the direction may be an upwards bias (overestimate the effect) or a downward bias (underestimate the effect). | Unpredictable |

Algorithm for reaching overall risk of bias judgement:

| Judgement | Interpretation | How reached |
| --- | --- | --- |
| *Low risk of bias except for concerns about uncontrolled confounding* | There is the possibility of uncontrolled confounding that has not been controlled for (given the observational nature of the study), but otherwise little or no concern about bias in the result | *Low risk of bias except for concerns about uncontrolled confounding* in Domain 1 and *Low risk of bias* in all other domains |
| *Moderate risk of bias* | There is some concern about bias in the result, although it is not clear that there is an important risk of bias | At least one domain is at *Moderate risk of bias*, but no domains are at *Serious risk of bias* or *Critical risk of bias* |
| *Serious risk of bias* | The study has some important problems: characteristics of the study give rise to a serious risk of bias in the result | At least one domain is at *Serious risk of bias*, but no domains are at *Critical risk of bias*  OR  Several domains are at *Moderate*, leading to an additive judgement of *Serious risk of bias* |
| *Critical risk of bias* | The study is very problematic: characteristics of the study give rise to a critical of bias in the result, such that the result should generally be excluded from evidence syntheses. | At least one domain is at *Critical risk of bias*  OR  Several domains are at *Serious risk of bias*, leading to an additive judgement of *Crticial risk of bias* |

This work is licensed under a [Creative Commons Attribution-NonCommercial-NoDerivatives 4.0 International License](http://creativecommons.org/licenses/by-nc-nd/4.0/).
