# Supplementary material for: Periprosthetic joint infection risk is comparable between unicompartmental knee arthroplasty to total knee arthroplasty conversion and primary total knee arthroplasty: A systematic review and meta‐analysis
Source: Knee Surg Sports Traumatol Arthrosc. 2025 Dec 8;34(9):3314–21. doi: 10.1002/ksa.70228 (PMC13502457; doi:10.1002/ksa.70228)
Supplement: Supplementary file 2 [file KSA-34-3314-s002.docx]

# **Supplementary material:**

Risk Of Bias In Non-Randomized Studies - of Interventions (ROBINS-I) Lunebourg

Risk Of Bias In Non-Randomized Studies - of Interventions (ROBINS-I) Cross

Risk Of Bias In Non-Randomized Studies - of Interventions (ROBINS-I) Järvenpää

Risk Of Bias In Non-Randomized Studies - of Interventions (ROBINS-I) Lim 2017

Risk Of Bias In Non-Randomized Studies - of Interventions (ROBINS-I) Lim 2019

Risk Of Bias In Non-Randomized Studies - of Interventions (ROBINS-I) Lombardi 2018

Risk Of Bias In Non-Randomized Studies - of Interventions (ROBINS-I) Risk of Bias assessment summary

Search Strategy/MeSH terms
