# Supplementary material for: Periprosthetic joint infection risk is comparable between unicompartmental knee arthroplasty to total knee arthroplasty conversion and primary total knee arthroplasty: A systematic review and meta‐analysis
Source: Knee Surg Sports Traumatol Arthrosc. 2025 Dec 8;34(9):3314–21. doi: 10.1002/ksa.70228 (PMC13502457; doi:10.1002/ksa.70228)
Supplement: Supplementary file 6 [file KSA-34-3314-s007.docx]

PubMed/Medline

(((conversion AND (UKA OR patellofemoral replacement OR patellofemoral arthroplasty OR ((unicompartmental OR Uni-compartmental OR Partial) AND (knee replacement OR knee arthroplasty OR &quot;Arthroplasty, Replacement, Knee&quot;[Mesh])) OR TKA OR total knee replacement OR total knee arthroplasty))) AND (&quot;Prosthesis-Related Infections&quot;[Mesh] OR periprosthetic joint infection OR Prosthetic joint infection OR Infection OR pji)) AND ((((1990:3000/12/12[pdat]) AND (english[LA])) OR (randomized controlled trial [pt] OR controlled clinical trial [pt] OR randomized [tiab] OR placebo [tiab] OR drug therapy [sh] OR randomly [tiab] OR trial [tiab] OR groups [tiab])) NOT (animals [mh] NOT humans [mh]))

Scopus

ALL ( ( conversion AND ( uka OR &quot;patellofemoral replacement&quot; OR &quot;patellofemoral arthroplasty&quot; OR ( ( unicompartmental OR uni-compartmental OR partial ) AND ( &quot;knee replacement&quot; OR &quot;knee arthroplasty&quot; OR &quot;Arthroplasty, Replacement, Knee&quot; ) ) OR tka OR &quot;total knee replacement&quot; OR &quot;total knee arthroplasty&quot; ) ) AND ( &quot;Prosthesis-Related Infections&quot; OR &quot;periprosthetic joint infection&quot; OR &quot;Prosthetic joint infection&quot; OR infection OR pji ) ) AND ( LIMIT-TO ( SUBJAREA , &quot;MEDI&quot; ) OR LIMIT-TO ( SUBJAREA , &quot;HEAL&quot; ) OR LIMIT-TO ( SUBJAREA , &quot;NURS&quot; ) )

Embase

(&#39;conversion&#39;/exp OR conversion) AND (uka OR &#39;patellofemoral replacement&#39; OR &#39;patellofemoral arthroplasty&#39;/exp OR &#39;patellofemoral arthroplasty&#39; OR ((unicompartmental OR &#39;uni compartmental&#39; OR partial) AND (&#39;knee replacement&#39;/exp OR &#39;knee replacement&#39; OR &#39;knee arthroplasty&#39;/exp OR &#39;knee arthroplasty&#39; OR &#39;arthroplasty, replacement, knee&#39;/exp OR &#39;arthroplasty, replacement, knee&#39;)) OR tka OR &#39;total knee replacement&#39;/exp OR &#39;total knee replacement&#39; OR &#39;total knee arthroplasty&#39;/exp OR &#39;total knee arthroplasty&#39;) AND (&#39;prosthesis-related infections&#39;/exp OR &#39;prosthesis-related infections&#39; OR &#39;periprosthetic joint infection&#39;/exp OR &#39;periprosthetic joint infection&#39; OR &#39;prosthetic joint infection&#39;/exp OR &#39;prosthetic joint infection&#39; OR &#39;infection&#39;/exp OR infection OR pji)
